# Inhibitors of Eicosanoid Biosynthesis Reveal that Multiple Lipid Signaling Pathways Influence Malaria Parasite Survival in *Anopheles gambiae*

**DOI:** 10.3390/insects10100307

**Published:** 2019-09-20

**Authors:** Hyeogsun Kwon, Ryan C. Smith

**Affiliations:** Department of Entomology, Iowa State University, Ames, IA 50011, USA; hskwon@iastate.edu

**Keywords:** eicosanoid signaling, mosquito, innate immunity, *Plasmodium*

## Abstract

Eicosanoids are bioactive signaling lipids derived from the oxidation of fatty acids that act as important regulators of immune homeostasis and inflammation. As a result, effective anti-inflammatory drugs have been widely used to reduce pain and inflammation which target key eicosanoid biosynthesis enzymes. Conserved from vertebrates to insects, the use of these eicosanoid pathway inhibitors offer opportunities to evaluate the roles of eicosanoids in less-characterized insect systems. In this study, we examine the potential roles of eicosanoids on malaria parasite survival in the mosquito *Anopheles gambiae.* Using *Plasmodium* oocyst numbers to evaluate parasite infection, general or specific inhibitors of eicosanoid biosynthesis pathways were evaluated. Following the administration of dexamethasone and indomethacin, respective inhibitors of phospholipid A2 (PLA2) and cyclooxygenase (COX), oocyst numbers were unaffected. However, inhibition of lipoxygenase (LOX) activity through the use of esculetin significantly increased oocyst survival. In contrast, 12-[[(tricyclo[3.3.1.13,7]dec-1-ylamino)carbonyl]amino]-dodecanoic acid (AUDA), an inhibitor of epoxide hydroxylase (EH), decreased oocyst numbers. These experiments were further validated through RNAi experiments to silence candidate genes homologous to EH in *An*. *gambiae* to confirm their contributions to *Plasmodium* development. Similar to the results of AUDA treatment, the silencing of EH significantly reduced oocyst numbers. These results imply that specific eicosanoids in *An. gambiae* can have either agonist or antagonistic roles on malaria parasite survival in the mosquito host.

## 1. Introduction

Mosquitoes are able to transmit numerous pathogens that cause high human morbidity and mortality [1,2], most notably malaria, which causes over 200 million cases and 435,000 deaths every year [3]. Caused by parasite of the genus *Plasmodium*, malaria is solely transmitted by anopheline mosquitoes [2,4]. Without a reliable vaccine, treatment has thus far relied on the administration of anti-malarial drugs, insecticides to reduce mosquito populations, and the use of long-lasting insecticidal nets (LLINs) to create a physical barrier for transmission. However, emerging drug and insecticide resistance has complicated current approaches of malaria control [2,5]. Therefore, understanding the mechanisms that influence malaria parasite survival in the mosquito host is critical for new strategies to control disease transmission.

*Plasmodium* parasites undergo severe bottlenecks in the mosquito which can be attributed to the innate immune response that limits parasite survival [4,6]. This includes multiple mechanisms of parasite killing [7,8,9,10,11,12,13] which involve the complex interplay between the mosquito midgut [14,15], immune cells known as hemocytes [12,13,16], and humoral components of the hemolymph [7,8,9]. However, our understanding of the mosquito immune responses that determine vectorial capacity remain limited. One aspect of invertebrate immunity that has thus far received little attention is the role of eicosanoids. These lipid-derived signaling molecules play critical roles in mediating inflammatory processes in vertebrates [17,18], yet have only recently been examined in insects through studies of immunity, renal physiology and reproduction [19,20,21,22,23,24,25].

Much of this work has been aided by commercially available eicosanoids and anti-inflammatory drugs which inhibit enzymes required for eicosanoid biosynthesis [26], enabling studies of eicosanoid function in insects [27,28,29]. Previous studies have demonstrated that eicosanoids have integral roles in immune function, mediating phagocytosis, encapsulation, and melanization responses to invading pathogens [28,29,30,31,32]. Yet, only recently has eicosanoid function begun to be addressed in the mosquito *Anopheles gambiae*, where prostaglandin and lipoxin synthesis has been implicated in anti-*Plasmodium* immunity and immune priming [23,25,33]. However, the study of eicosanoids in mosquito immunity has been impaired by the lack of characterization of key oxidative enzymes required for the conversion of arachidonic acid (AA) to prostaglandins (PGs), leukotrienes (LTs), lipoxins (LXs) and dihydroxyeicosatrienoic acids (DHETs) derivatives.

Therefore, this study aims to address potential roles of eicosanoids in *Plasmodium* survival by the administration of anti-inflammatory drugs targeting each of the major eicosanoid biosynthesis pathways. Results from these inhibition experiments demonstrate that specific eicosanoids, and the downstream effects of their activation, can behave as agonists or antagonists of malaria parasite survival in the mosquito host. Together, these results argue that eicosanoids are important mediators of mosquito physiology, and capable of influencing vectorial capacity in *An. gambiae*.

## 2. Materials and Methods

### 2.1. Mosquito Rearing

*Anopheles gambiae* mosquitoes (Keele strain) were reared at 27 °C and 80% relative humidity, with a 14/10 hour day/night cycle. Larvae were fed on fish flakes (Tetramin, Tetra), and adult mosquitoes were maintained on 10% sucrose solution.

### 2.2. Eicosanoid Inhibitors

Dexamethasone ((11β,16α)-9-Fluoro-11,17,21-trihydroxy-16-methylpregna-1,4-diene-3,20-dione) a phospholipase A2 inhibitor, indomethacin (1-(4-Chlorobenzoyl)-5-methoxy-2-methyl-3-indoleacetic acid) a cyclooxygenase (COX) inhibitor, esculetin (6,7-Dihydroxycoumarin) a lipoxygenase (LOX) inhibitor, and AUDA (12-[[(tricyclo[3.3.1.13,7] dec-1-ylamino) carbonyl]amino]-dodecanoic acid) an epoxide hydrolase inhibitor, were purchased from Sigma-Aldrich. The chemicals were dissolved in 100% ethanol and diluted in 1 × PBS (phosphate buffered saline, pH 7.4) to prepare 10 μg/μL working solution (23 mM of dexamethasone, 28 mM of indomethacin, 110 mM of esculetin and 12 mM of AUDA in 1× PBS) similar to previous experiments [34]. Before initial use, inhibitor stocks were heated at 75 °C for 5 min to ensure that samples were completely dissolved. Sixty-nine nanoliters (nL) of each inhibitor or 1× PBS: 5% ethanol control was injected intra-thoracically into naïve female mosquitoes (3- to 5-day old) and maintained at 19 °C prior to subsequent challenge experiments.

### 2.3. *Plasmodium berghei* Infections

Female Swiss Webster mice were infected with a mCherry strain of *Plasmodium berghei* as described previously [12,13]. For inhibitor experiments, mosquitoes were challenged with an infected mouse 24 h post-injection of either 1× PBS: 5% ethanol (control) or each of the respective eicosanoid inhibitors. To evaluate the effects of gene-silencing on malaria parasite infection, mosquitoes were challenged with a *P. berghei*-infected mouse four days post-injection of dsRNA. Infected mosquitoes were maintained at 19 °C until mosquitoes were dissected in 1× PBS seven or eight days post-infection to determine oocyst numbers from individual mosquito midguts by fluorescence microscopy using a Nikon Eclipse 50i (Nikon, Tokyo, Japan).

### 2.4. Gene-Silencing Experiments

RNAi experiments were performed as previously described [12,13]. Briefly, T7 primers were designed using the E-RNAi web application (http://www.dkfz.de/signaling/e-rnai3/idseq.php) to specifically target genes of interest. These are listed in Table S1. cDNA prepared from whole mosquitoes collected 24 h post-*P. berghei* infection was used as a template for dsRNA synthesis. PCR amplicons were gel purified using the Gel DNA Recovery kit (Zymo Research, Orange, CA, USA) and dsRNA was prepared using the MEGAscript RNAi kit (Life Technologies, NewYork, NY, USA) according to the manufacturer’s instructions. dsRNA was resuspended in nuclease free water to a final concentration of 3 µg/µL. Three to four day old mosquitoes were cold anesthetized and injected intrathoracically with 69 nL (~200 ng) of dsRNA per mosquito using a Nanoject III injection system (Drummond Scientific).

The effects of gene-silencing were evaluated four days post-injection of dsRNA in whole mosquito samples (~15 mosquitoes per treatment). Total RNA was isolated using TRIzol (Thermo Fisher Scientific, Waltham, MA, USA) and then treated with DNase I to remove gDNA contamination according to the manufacture’s protocol (New England Biolabs, Ipswich, MA, USA). Total RNA (2 µg) was used for cDNA synthesis using the RevertAid First Strand cDNA Synthesis kit (Thermo Fisher Scientific), with resulting cDNA (1:5 dilution) used as a template for amplification with 500 nM of gene-specific primers. Ribosomal protein S7 transcript levels were used as an internal reference as previously [11,12,13]. qRT-PCR was performed using PowerUp™SYBR^®^Green Master Mix (Thermo Fisher Scientific) with the following cycling conditions: 95 °C for 10 min, 40 cycles with 95 °C for 15 s and 65 °C for 60 s. A comparative C_T_ (2^−ΔΔCt^) method was employed to determine relative transcript abundance for each transcript [35].

### 2.5. Gene Expression Analysis

To determine whether silencing epoxide hydrolase was linked to the regulation of anti-microbial peptide (AMPs) expression, relative AMP transcripts were examined using cDNA samples and qRT-PCR experiments as described above. A list of primers used for gene expression analyses are listed in Table S1.

## 3. Results

**Figure 1:**
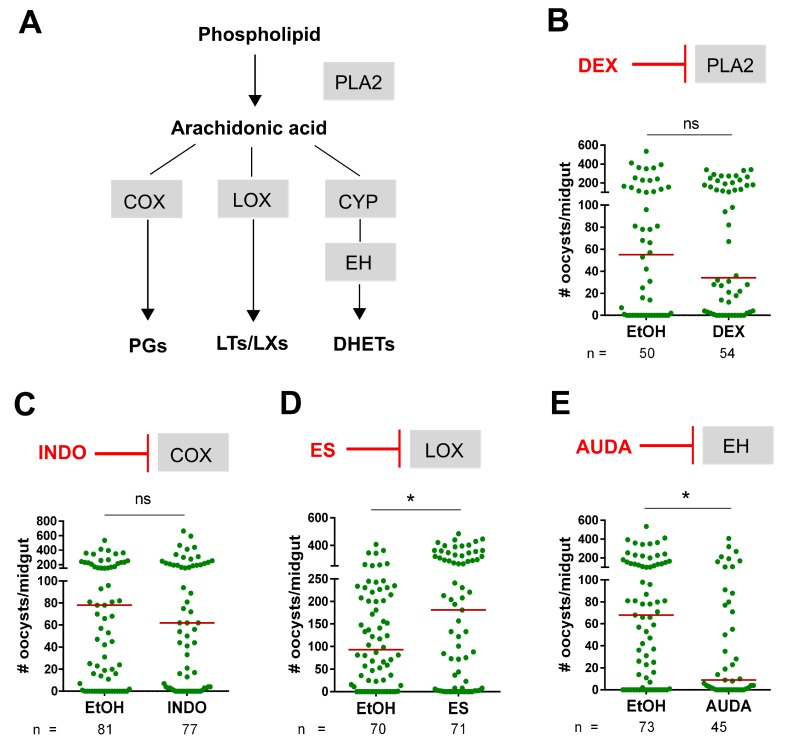
Effect of eicosanoid inhibitors on *Plasmodium* oocyst survival. Biosynthesis pathways of eicosanoids as described in mammalian systems are summarized in (**A**). Arachidonic acid (AA) is derived from membrane phospholipids by hydrolysis of phospholipase A2 (PLA2) activity. The AA is further metabolized into various eicosanoids, such as prostaglandins (PGs), leukotrienes (LTs), lipoxins (LXs) and dihydroxyeicosatrienoic acids (DHETs) through the activity of specific enzymes, cyclooxygenase (COX), lipoxygenase (LOX), cytochrome P450 (CYP), and epoxide hydrolase (EH), respectively. The influence of specific eicosanoid inhibitors on *P. berghei* survival was addressed in mosquitoes injected with each respective inhibitor by examining oocyst numbers at 8 days post-infection (**B**–**E**). Compared to an ethanol (EtOH) control, oocyst survival was examined for the PLA2 inhibitor, dexamethasone (DEX) (**B**), a COX inhibitor, indomethacin (INDO) (**C**), a LOX inhibitor, esculetin (ES) (**D**), and an epoxide hydrolase inhibitor, AUDA (**E**). Data were analyzed by Mann-Whitney U using GraphPad Prism 6.0. Bar graphs represent mean ± SEM of three independent experiments. Asterisks denote significance (* *p* < 0.05); ns, not significant.

Previous work has suggested that eicosanoids contribute to the production of anti-microbial peptides, immune priming, and anti-*Plasmodium* immunity in *Anopheles* [23,25,33]. However, the characterization of the key oxidative enzymes converting arachidonic acid into specific eicosanoids has been limited in mosquitoes [25,36]. To overcome this hurdle, we examined the potential role of eicosanoids on *Plasmodium* infection using known eicosanoid inhibitors in *An. gambiae*. Prior to taking an infected blood meal, mosquitoes were primed by the injection of specific inhibitors to interrupt the conversion of arachidonic acid (AA) to prostaglandins (PGs), leukotrienes (LTs), lipoxins (LXs), or dihydroxyeicosatrienoic acid (DHETs) derivatives (Figure 1A) to determine their respective influence on malaria parasite survival. When PLA2 was targeted by dexamethasone treatment (Figure 1B) or when cyclooxygenase (COX) activity was impaired by indomethacin (Figure 1C), *Plasmodium* oocyst numbers were unchanged when compared to control mosquitoes (Figure 1B,C). However, treatment with esculetin or AUDA significantly influenced parasite survival with agonistic or antagonistic effect. Esculetin treatment significantly increased oocyst numbers (Figure 1D), while AUDA treatment decreased *Plasmodium* survival (Figure 1E). Together, these results suggest that leukotrienes/lipoxins and DHETs influence mosquito immunity and/or physiology to mediate vectorial capacity.

**Figure 2:**
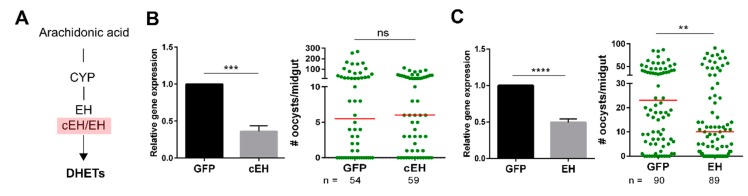
Effect of putative genes involved in dihydroxyeicosatrienoic acid (DHETs) biosynthesis on *Plasmodium* oocyst survival. By summarizing recent studies on functional characterization of insect orthologous genes to human epoxide hydrolase (EH), orthologous genes (cEH and EH in pink boxed) in *An. gambiae* were selected for RNAi experiments (**A**). The efficiency of gene-silencing was verified by qRT-PCR and the effects of the gene knockdown on oocyst survival were evaluated for either cytoplasmic epoxide hydrolase (cEH) (**B**) or EH (**C**) and compared to green fluorescent protein (GFP) controls. qRT-PCR data were analyzed by an unpaired t-test. Bar graphs represent mean ± SEM of three independent experiments. Data from oocyst assessment were pooled from three or more independent experiments with statistical analysis determined by a Mann–Whitney test using GraphPad Prism 6.0. Asterisks denote significance (** *p* < 0.01, *** *p* < 0.001, **** *p* < 0.0001); ns, not significant. CYP, cytochrome P450.

Since previous work has argued for the role of lipoxins in immune priming and innate immune function in *An. gambiae* [23], and genes orthologous to mammalian lipoxygenase (LOX) have not been found, we focused our attention on the DHET pathway. Using orthologous genes for epoxide hydrolase [36,37,38], the enzyme responsible for the production of DHETs (Figure 2A), we identified two potential candidates in the *An. gambiae* genome: cytoplasmic epoxide hydrolase 2 (cEH2; AGAP003542) and epoxide hydrolase (EH; AGAP011972), of which EH function has previously been confirmed for the generation of epoxy fatty acids [36]. Thus, RNAi experiments targeting each gene were performed to determine their roles in *Plasmodium* survival. Both cEH and EH were effectively silenced following dsRNA injection as compared to GFP controls (Figure 2B,C), yet only EH-silencing significantly influenced oocyst numbers (Figure 2C), resulting in a similar phenotype to AUDA treatment (Figure 1E). This provides further support for the influence of DHETs on malaria parasite survival.

**Figure 3:**
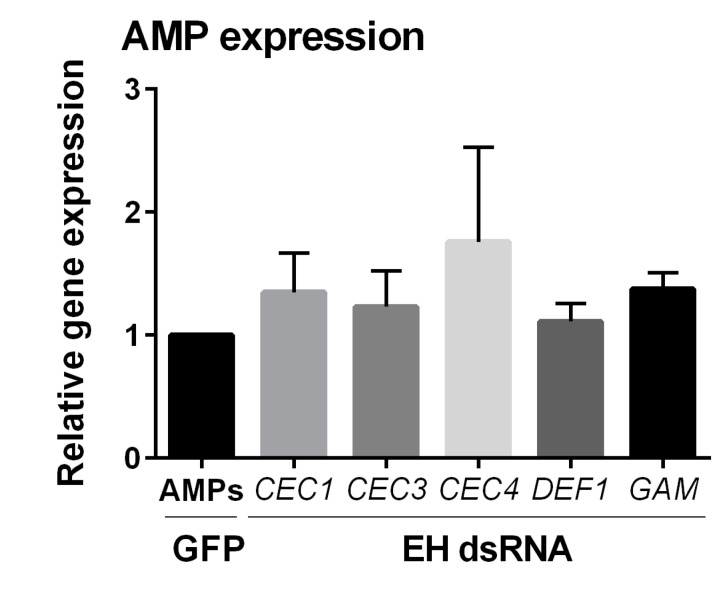
Epoxide hydrolase (EH)-silencing does not influence AMP expression. The effects of EH-silencing on anti-microbial peptide (AMP) expression were evaluated by qRT-PCR. Relative gene expression was compared between EH-silenced whole mosquitoes and that of green fluorescent protein (GFP) controls. Data were analyzed using an unpaired t-test to determine differences in relative gene expression of each respective AMP gene between GFP and EH dsRNA treatments. Bars represent mean ± SEM of three independent experiments. CEC1 (cecropin 1, AGAP000693), CEC3 (cecropin 3, AGAP000694), CEC4 (cecropin 4, AGAP006722), DEF1 (defensin 1, AGAP011294) and GAM (gambicin, AGAP008645).

To further examine the influence of DHETs in *An. gambiae*, we examined the gene expression of anti-microbial peptides (AMPs). Previous studies have argued that the ingestion of AUDA in *Culex quinquefasciatus* increased AMP production [38], therefore we wanted to determine if increased AMP production could similarly account for the reduction in *Plasmodium* survival following AUDA-treatment (Figure 1E) that were reconstituted by EH-silencing (Figure 2C). To approach this question, orthologs of the AMPs previously examined in response to the ingestion of AUDA in *Culex* were evaluated; including cecropin 1 (CEC1, AGAP000693), cecropin 3 (CEC3, AGAP000694), cecropin 4 (CEC4, AGAP006722), defensin 1 (DEF1, AGAP011294) and gambicin (GAM, AGAP008645). However, when AMP expression was examined after EH-silencing, no discernable differences were detected (Figure 3). This suggests that the negative influence of AUDA-treatment and EH-silencing on malaria parasite survival is independent of AMP expression.

## 4. Discussion

Eicosanoids have been extensively studied in mammalian systems for their roles in immune regulation and inflammation, by modulating cytokine release, cell differentiation, cell migration, antigen presentation, and apoptosis [18,39,40]. Similar conserved responses have also been described in insects, with eicosanoids mediating cellular immune function through phagocytosis, encapsulation, or melanization responses [28,29,30,31,32]. Recent studies have also implicated eicosanoid function in mosquito immune priming to subsequent pathogen challenge [23,25]. However, the significance of eicosanoids in mosquito physiology and immune function has only recently been addressed [23,25,33,36,37,38,41], leaving several fundamental questions of eicosanoid function in the mosquito host unanswered. This includes the potential role of eicosanoids in mediating the vectorial capacity of *Anopheles* to malaria parasites [23,25,41], which, due to the complexity of eicosanoid pathways and the limited knowledge of the enzymes responsible for its biosynthesis, has not been fully addressed. Using a pharmaceutical approach with known eicosanoid inhibitors, we examined the potential roles of each major eicosanoid biosynthesis pathway on *Plasmodium* oocyst numbers. 

The initiation of eicosanoid biosynthesis is elicited by phospholipase A2 (PLA2) activity, which cleaves fatty acid and subsequently releases arachidonic acid (AA) [42]. In insects, the important roles of PLA2 in hemocyte migration [25,43] and antimicrobial peptide (AMP) expression [33,44,45] have been described following the injection of the PLA2 inhibitors, dexamethasone and benzylideneacetone. However, when mosquitoes were treated with dexamethasone prior to challenging with *P. berghei*, malaria parasite numbers were not affected. Given the reciprocal outcomes of esculetin and AUDA treatment which respectively increase and decrease parasite survival, we argue that the presumed downstream influence on each of these respective pathways likely counter the singular effects of each pathway.

Similar to dexamethasone, our experiments with indomethacin do not influence *Plasmodium* oocyst numbers, suggesting that prostaglandins do not contribute to parasite survival. However, this contrasts a recent report which states that mosquito anti-*Plasmodium* immunity can be primed by the injection of several prostaglandin derivatives including PGE1, PGE2 and PGF2α [25]. One possible explanation is that indomethacin, a COX inhibitor, may not inhibit the heme peroxidases HPX7 and HPX8 which were recently implicated in PGE2 synthesis [25]. Alternatively, since indomethacin can impair vertebrate peroxidase activity [46], and the injection of indomethacin can influence *Plasmodium* sporozoite salivary gland numbers [41], it is plausible that indomethacin can similarly inhibit mosquito HPX activity. Further experiments are therefore required to delineate the potential inhibitory effects of indomethacin on prostaglandin signaling in mosquitoes.

Our experiments with esculetin, a specific inhibitor of LOX, suggest that an unidentified enzyme(s) analogous to lipoxygenases could be present in *An. gambiae.* Esculetin treatment prior to *P. berghei* infection resulted in a significant increase in oocyst numbers, suggesting that the inhibition of leukotriene and/or lipoxin synthesis makes mosquitoes more susceptible to infection. These findings are in agreement with previous work demonstrating that lipoxin-A4 and -B4 mediate immune priming [23].

In addition, our experiments demonstrate that AUDA treatment can significantly reduce parasite survival. Previous studies in *An. gambiae* have suggested that AUDA inhibits the enzymatic activity of an epoxide hydrolase which metabolizes epoxide fatty acids [36]. In our experiments the silencing of *EH*, but not *cEH*, recapitulate the effects of AUDA on *Plasmodium* numbers. This suggests that impairing the production of downstream DHETs influences mosquito immunity or physiology, making mosquitoes more refractory to parasite infection. Based on the previously described increase in AMP expression following AUDA ingestion [38], we similarly examined AMP expression in *EH*-silenced mosquitoes to see if this could account for the increased anti-*Plasmodium* effects associated with this phenotype. However, there was no change in AMP expression, leaving the molecular mechanisms underlying EH enzymatic activity and its influence on parasite survival unresolved in this study. At present, it is unclear if these effects are immune-related or due to physiological changes which limit resources required for *Plasmodium* development in the mosquito host.

## 5. Conclusions

Through these studies, we have addressed the role of eicosanoid biosynthesis on malaria parasite survival through the use of pharmaceutical inhibitors, which offer new potential areas of investigation into the lipid signaling mechanisms that determine mosquito vector competence. However, the molecular contributions of eicosanoids on *Plasmodium* development still remain elusive, primarily due to the incomplete understanding of the core enzymes involved in eicosanoid biosynthesis and the resulting downstream effects of lipid signaling on mosquito innate immunity and physiology. As a result, we believe that this study is an important initial step in our understanding of eicosanoid function in mosquitoes.

## Supplementary Materials

The following are available online at https://www.mdpi.com/2075-4450/10/10/307/s1, Table S1: Primers used for dsRNA synthesis and qRT-PCR.
