# Supplementary material for: Inhibitors of Eicosanoid Biosynthesis Reveal that Multiple Lipid Signaling Pathways Influence Malaria Parasite Survival in Anopheles gambiae
Source: Insects. 2019 Sep 20;10(10):307. doi: 10.3390/insects10100307 (PMC6835628; doi:10.3390/insects10100307)
Supplement: Supplementary file 1 [file insects-10-00307-s001.pdf]

**Table S1. Primers used for dsRNA synthesis and qRT-PCR**

| Primer name | Sequences | Orientation | Gene ID |
|---|---|---|---|
| **For dsRNA synthesis** | | | |
| Anoga-EHT7F | 5'-TAATACGACTCACTATAGGGGACACCGAGAAGCCCCAGTA-3' | Sense | AGAP011972 |
| Anoga-EHT7R | 5'-TAATACGACTCACTATAGGGACCTCGAACAGGTGGAAGTC-3' | Antisense | |
| Anoga-cEH2T7F | 5'-TAATACGACTCACTATAGGGGATCTGGGGGCAACGATT-3' | Sense | AGAP003542 |
| Anoga-cEH2T7R | 5'-TAATACGACTCACTATAGGGCCGTTTGGCAGAAGCTGTA-3' | Antisense | |
| **For qRT-PCR** | | | |
| Anoga-EHqF | 5'-AATCTGGAGTTCCGTGTCGT-3' | Sense | AGAP011972 |
| Anoga-EHqR | 5'-GCCCTCACATTGAGCTACAC-3' | Antisense | |
| Anoga-cEH2qF | 5'-CGCAACTTTCTGTTCTACCGT-3' | Sense | AGAP003542 |
| Anoga-cEH2qR | 5'-GCCACACTGCTCGATGATTT-3' | Antisense | |
| Anoga-CEC1qF | 5'-CAGCAGAGAAGGCCCTACCG-3' | Sense | AGAP000693 |
| Anoga-CEC1qR | 5'-TCATGTTAGCAGAGCCGTCGT-3' | Antisense | |
| Anoga-CEC3qF | 5'-ACGTACTGAACCACCTGCGCGTT-3' | Sense | AGAP000694 |
| Anoga-CEC3qR | 5'-GCGCTGTGTGCGCCGATGAA-3' | Antisense | |
| Anoga-CEC4qF | 5'-CCACGCTGCTACTGTTCGGT-3' | Sense | AGAP006722 |
| Anoga-CEC4qR | 5'-CTGCAGTACGGGCACTACCT-3' | Antisense | |
| Anoga-GAMqF | 5'-AGTGGAGCTCTGCTGGAACC-3' | Sense | AGAP008645 |
| Anoga-GAMqR | 5'-TCGTAAACTGCACCGCACTG-3' | Antisense | |
| Anoga-rps7qF | 5'-ACCACCATCGAACACAAAGTTGACACT-3' | Sense | AGAP010592 |
| Anoga-rps7qR | 5'-CTCCGATCTTTCACATTCCAGTAGCAC-3' | Antisense | |
